# Impact of chronic diuretic treatment on glucose homeostasis

**DOI:** 10.1186/1758-5996-5-80

**Published:** 2013-12-13

**Authors:** Silvio Buscemi, Antonio Nicolucci, Giuseppe Lucisano, Fabio Galvano, Giuseppe Grosso, Fatima M Massenti, Emanuele Amodio, Alice Bonura, Delia Sprini, Giovam B Rini

**Affiliations:** 1 Dipartimento Biomedico di Medicina Interna e Specialistica (DIBIMIS) – Laboratorio di Nutrizione Clinica, University of Palermo, Via del Vespro, 129, Palermo 90127, Italy; 2 Dipartimento di Farmacologia Clinica ed Epidemiologia, Consorzio Mario Negri Sud, Via Nazionale per Lanciano, 8, S. Maria Imbaro, Chieti, Italy; 3 Dipartimento di Scienze del Farmaco, University of Catania, viale Andrea Doria,6, Catania 95124, Italy; 4 Dipartimento di Scienze per la Promozione della Salute e Materno Infantile, University of Palermo, Via del Vespro, 129, Palermo 90128, Italy

**Keywords:** Diuretics, Hypertension, Insulin resistance, Type 2 diabetes, Uric acid

## Abstract

**Background:**

The use of diuretics for hypertension has been associated with unfavorable changes in cardiovascular risk factors, such as uric acid and glucose tolerance, though the findings in the literature are contradictory.

**Methods:**

This study investigated whether diuretic use is associated with markers of metabolic and cardiovascular risk, such as insulin-resistance and uric acid, in a cohort of adults without known diabetes and/or atherosclerotic cardiovascular disease. Nine hundred sixty-nine randomly selected participants answered a questionnaire on clinical history and dietary habits. Laboratory blood measurements were obtained in 507 participants.

**Results:**

Previously undiagnosed type 2 diabetes was recognized in 4.2% of participants who were on diuretics (n = 71), and in 2% of those who were not (n = 890; P = 0.53). Pre-diabetes was diagnosed in 38% of patients who were on diuretics, and in 17.4% (P < 0.001) of those who were not. Multivariate analysis showed that insulin-resistance (HOMA-IR) was associated with the use of diuretics (P = 0.002) independent of other well-known predisposing factors, such as diet, physical activity, body mass index, and waist circumference. The use of diuretics was also independently associated with fasting plasma glucose concentrations (P = 0.001) and uric acid concentrations (P = 0.01).

**Conclusions:**

The use of diuretics is associated with insulin-resistance and serum uric acid levels and may contribute to abnormal glucose tolerance.

## Background

Hypertension affects up to 60% of patients with type 2 diabetes [1]. It has been reported that the concomitance of hypertension and diabetes roughly doubles the risk of cardiovascular events [2,3]. Indeed, treatment of hypertension with diuretics has often been attributed to increased insulin resistance and accelerated onset of diabetes [4-7]. It has also been reported that hypertension often precedes the onset of diabetes, suggesting that anti-hypertensive treatment with diuretics may contribute to the development of abnormal glucose tolerance, thus offsetting the benefits of the treatment in terms of cardiovascular risk [8,9]. Furthermore, the use of diuretics, particularly thiazide, has also been associated with unfavorable alterations in other cardiovascular risk factors, such as uric acid and cholesterol concentrations [10-13].

Despite these negative effects, it has been recommended that treatment of hypertension be prioritized and stressed in persons with type 2 diabetes, where first choice agents may include thiazide diuretics [14]. In fact, the trend of the use of diuretics, especially that of thiazides, is continually increasing [15]. Present guidelines indicate thiazides as first-line therapy in hypertension [16], but also suggest that their use as first step drugs be limited in the treatment of diabetic patients [17]. Furthermore, the impact of anti-hypertensive treatment with diuretics on insulin resistance and glycemia in people without known diabetes and/or atherosclerotic diseases is still a matter of debate [14,18-21].

Therefore, we investigated the associations between diuretic treatment for hypertension and different markers of metabolic and cardiovascular risk in a cohort of randomly selected adults without known diabetes and/or atherosclerotic cardiovascular disease.

## Methods

This observational, cross-sectional study was carried out in Palermo, the largest city in Sicily, Italy, with a population of 663,173 from March 28th to April 10th, 2011. Groups composed of physicians (n = 5) and dieticians (n = 13) alternated their presence inside the *Forum*, a shopping mall in Palermo, from 9:00 a.m. until 9:00 p.m. There they contacted those customers who asked to participate in the study, which had been proposed by means of posters at the mall.

The *Forum* is the largest shopping center in Palermo, and customers come from all parts of the city, suburbs and neighboring areas. Data provided by the *Forum* administration show that the characteristics of their habitual customers were heterogeneous in terms of gender (female 65%, male 35%), age (10–54 years 50%, > 55 years 50%), place of residence (Palermo 62%, outside of Palermo 38%), education (college graduates = 14%, high school graduates = 37%, middle school = 32%, primary school = 17%), and employment status (housewife = 40%, retired = 23%, employed = 19%, student = 8%, unemployed = 6%, manager/professional = 4%).

Inclusion criteria were age ≥ 18, and residence in the province of Palermo. Exclusion criteria were gastrointestinal or connective diseases, chronic pancreatitis, liver cirrhosis, use of nonsteroidal antiinflammatory drugs, corticosteroids, or drugs interfering with coagulation; pregnancy or lactation in the past 6 months. In order to encourage the participation of younger people without known cardiovascular, metabolic or nutritional diseases, an echographic check of the thyroid was also proposed.

There was no incentive provided to the participants. Participants were asked to present, in the morning, in overnight fasting conditions, at the Biomedical Department of the Internal and Specialized Medicine’s Laboratory of Clinical Nutrition, at the University of Palermo, in the following weeks, and no later than July 15th, 2011, to undergo blood sampling for assessment of blood chemistry and hormone values. A blood sample was frozen and stored at −80°C, and a sample was treated and stored for subsequent measurements.

Our institutional ethics committee at the Biomedic Department of Internal and Specialistic Medicine approved the study protocol. Each participant signed an approved informed consent form.

Participants were administered a questionnaire on demographic characteristics, the presence of chronic disease and pharmacologic treatment, physical activity, including items concerning the level of physical activity and its weekly frequency, daily time watching television, on the computer, and playing video games. Physical activity was classified as follows: none = no significant active physical activity (most of the time spent sitting at home or at work; light = short walks (including at work or at home, walking from place to place, and any other walking done for recreation, exercise, or leisure for 10–20 minutes/day.); moderate = sports activity, including fast walking or bicycling for 20 minutes/day 1–3 times a week; heavy = sports activity, including fast walking or bicycling for > 20 minutes/day or heavy work activities > 3 times a week). Half-quantitative habitual intakes of different foods during the past 12 months were assessed with the Food Frequency Questionnaire (FFQ) [22]. The requested information referred to the last year. With a different analysis in the same cohort, using an *a posteriori* approach, we applied a cluster analysis to identify dietary patterns [23], a procedure that is based on the intercorrelations among food groups or nutrients. A diet that could be defined as unhealthy was identified, and was characterized, by high consumption of soft drinks, fried foods, seed oils, cured meats, butter, red meat and sweets; a dietary pattern that resembled the Mediterranean diet, defined as healthy, was characterized by high intakes of fruit, milk and cheese, olive oil, vegetables, pasta and bread; a third pattern of dietary habits was defined as intermediate, and had characteristics that were between the two other diets. Type 2 diabetes and pre-diabetes were defined according to the most recent consensus statements [24]. In particular, previously undiagnosed type 2 diabetes was defined on the basis of a fasting plasma glucose concentration of >125 mg/dl and/or random capillary blood glycemia >199 mg/dl and/or glycated hemoglobin >6.4%. Pre-diabetes was diagnosed when the fasting plasma glucose concentration was in the range 100–125 mg/dl and/or glycated hemoglobin between 5.7-6.4%. The habitual use of anti-hypertensive drugs was investigated and questions on the use of diuretics (hydrochlorotiazides, furosemide, spironolactone), beta-blockers, angiotensin converting enzyme inhibitors (ACEI) or angiotensin receptor blockers (ARBs), calcium channel antagonists (CCA), alpha-blockers, and clonidine were categorized as follows: no habitual consumption = 0, habitual consumption = 1.

### Measurements

Height and body weight were measured with participants lightly dressed and without shoes (SECA); the body mass index (BMI) was calculated as body weight (kg)/height^2^ (m^2^). Body circumferences were measured at the umbilicus (waist circumference) and at the most prominent buttock level (hip circumference).

Systolic and diastolic arterial blood pressure (two measurements obtained at 5-minute intervals in seated position) and heart rate were measured by physicians or dietitians with an oscillometric device, and according to standardized procedures (Omron M6; Omron Healthcare Co; Matsusaka, Mie, Japan).

### Laboratory analysis

Capillary blood glucose concentrations were randomly assessed using a glucose reflectometer (Glucocard G meter; Menarini Diagnostics; Florence, Italy). Fasting plasma glucose (FPG), total cholesterol, high-density lipoproteins (HDL) cholesterol, triglycerides, uric acid and creatinine concentrations were ascertained with standard clinical chemistry methods (Glucosio HK UV; Colesterolo tot. Mod P/D; Colesterolo HDL gen 3 mod P/917; Trigliceridi; Acido urico MOD P/917; Creatinina enzimatica; Roche diagnostics, Monza, Italy). Basal insulin concentrations (Elecsys insulina; Roche diagnostics; Monza, Italy) and glycated hemoglobin (HbA_1_c; HbA_1_c gen.3; Roche diagnostics; Monza, Italy) were also measured. Low-density lipoprotein (LDL) cholesterol concentration was calculated by means of Friedewald’s formula [25]. Estimated glomerular filtration rate (eGFR) was calculated according to modification of diet in renal disease study (MDRD) [26].

Both the homeostasis model assessment of insulin resistance (HOMA-IR) and the homeostasis model assessment of β-cell function (HOMA-β) were calculated as defined by Matthews et al. [27].

### Statistical analysis

Patient baseline characteristics are reported as frequency (percentage) and mean ± SD or median along with lower and upper quartiles.

Linear regression analyses were done to evaluate factors associated with HOMA-IR, HOMA-β, fasting plasma concentrations of glucose, and uric acid. The following baseline covariates were tested: age, gender, BMI, waist circumference, dietary pattern (Healthy, Intermediate, Unhealthy), level of physical activity (light, moderate/heavy or none), use of diuretics (yes or no), and use of beta-blockers (yes or no). A two-tailed Wald chi-squared P value of < 0.05 was considered significant. Multivariate logistic regression analysis was done to evaluate factors associated with pre-diabetes, and the following baseline covariates were tested: age, gender (male vs. female), BMI, dietary pattern, physical activity level, use of diuretics (yes or no), and use of beta-blockers (yes or no).

All statistical analyses were done using SAS version 9.2 (SAS Institute Inc; Cary, NC, US).

## Results

A total of 1,231 (465 males and 766 females) participants were evaluated; 270 participants were excluded because of the presence of diabetes (type 1 or 2), clinically known atherosclerotic diseases (coronary heart disease, previous stroke, carotid or peripheral atherosclerosis), chronic renal failure or incomplete anthropometric measurements. Laboratory blood measurements were obtained in 507 participants.

Males constituted 38.2%, 20% of the cohort were current smokers, and 37.3% habitually consumed alcohol (at least 5 glasses of wine or beer or superalcoholic a week). The different dietary patterns of the participants were as follows: Mediterranean diet = 34.2% (n = 329), Unhealthy = 20.8% (n = 200) and Intermediate diet = 45.0% (n = 432). Habitual physical activity was classified as “none” in 456 participants (47.4%), “light” in 356 participants (37.0%) and “moderate/heavy” in 149 participants (15.6%). The frequency of known hypertension was 27.2%. The use of anti-hypertensives was as follows: diuretics = 7.4%, beta-blockers = 8.6%, ACEI/ARBs = 15.5%, CAA = 3.4%. The classes of diuretics used were as follows: hydrochlorothiazide in 83.1% of patients on diuretic treatment (maximum reported daily dose = 25 mg), furosemide in 11.3% of cases (maximum reported daily dose = 25 mg), and spironolactone in 5.6% (maximum reported daily dose = 50 mg). Statins were regularly assumed by 7.1% of participants, and ω-3 fatty acids by 1.6%. Previously undiagnosed type 2 diabetes was recognized in 2.2%, and pre-diabetes in 19.7% of the cohort.

Demographic, anthropometric and clinical characteristics of the participants are reported in Table 1. Previously undiagnosed type 2 diabetes was identified in 3 (4.2%), pre-diabetes in 27 (42.2%) of participants with hypertension who were on diuretics (n = 71), and in 28.9% (6 type 2 diabetics and 49 pre-diabetics) of those with hypertension who were not (n = 190; P = 0.03). The percentage of males was not significantly different between participants who were on diuretics (38%), and those who were not (36.0%; P = 0.65). The metabolic characteristics of participants according to the use of diuretics or not are presented in Table 2. The predictors of HOMA-IR, HOMA-β, FPG and uric acid concentrations, identified by multiple logistic regression analysis, are reported in Tables 3, 4, 5, and 6. In particular, the use of diuretics was unfavorably associated with HOMA-IR (P = 0.002), FPG (P = 0.001) and uric acid plasma concentrations (P = 0.01). No significant association was observed with the use of beta-blockers. The predictors of HbA_1_c were gender (male = 1; estimate = 0.08; P < 0.05), age (estimate = 0.01; P < 0.001) and BMI (estimate = 0.01; P < 0.05). Male gender (estimate = −100; P < 0.001) and BMI (estimate = −0.56; P < 0.005) were the only significant predictors of HDL-cholesterol blood concentrations. Triglyceride levels were significantly predicted by Healthy (estimate = −154) or Intermediate (estimate = −132) dietary patterns (P < 0.05), gender (male = 1; estimate = 118; P < 0.05), and BMI (estimate = 14.7; P < 0.05). No significant association was found among ACEI/ARBs, CAA and other variables considered in this study. Multivariate logistic regression analysis demonstrated that only age and waist circumference were significantly associated with pre-diabetes (Table 7).

**Table 1:** Clinical and biochemical characteristics of the 961 participants

	**Mean ± SD**	**Median (Q1-Q3)**
Age (y)	49 ± 14	50 (38–60)
Body weight (kg)	73.6 ± 15.9	72.0 (61.6 - 83.5
BMI (kg/m^2^)	27.9 ± 5.4	27.2 (24.1-31.0)
Circumferences		
Waist (cm)	94.3 ± 14.5	94.0 (85.0 - 103.0)
Hip (cm)	104.6 ± 12.3	104.0 (98.0 - 110.0)
Systolic BP (mmHg)	129 ± 16	128 (118–139)
Diastolic BP (mmHg)	79 ± 10	78 (72–85)
Heart rate (beats/min)	74 ± 11	73 (66–81)
Random capillary blood glucose (mg/dl)	91 ± 12	90 (82–97)
Blood concentration of	n = 507	
glycated hemoglobin%	5.6 ± 0.4	5.5 (5.3 - 5.8)
mmol/mol	37.4 ± 4.8	36.6 (34.4 - 39.9)
glucose (mg/dl)	88 ± 21	85 (75–97)
total cholesterol (mg/dl)	213 ± 39	212 (185–240)
hdl cholesterol (mg/dl)	59 ± 15	58 (49–67)
triglycerides (mg/dl)	101 ± 49	90 (68–121)
ldl cholesterol (mg/dl)	133 ± 36	131 (108–161)
uric acid (mg/dl)	5.0 ± 1.4	4.7 (3.9 - 5.9)
insulin (μu/ml)	9.6 ± 5.8	8.2 (5.6 - 11.8)
HOMA-IR	2.2 ± 1.5	1.8 (1.2 - 2.7)
HOMA-β	141.8 ± 104.5	117.9 (80.0 - 171.4)
Creatinine (mg/dL)	0.83 ± 0.21	0.79 (0.69 - 0.94)
eGFR-MDRD (mL/min/1.73 m^2^)	91.8 ± 20.3	90.3 (76.5 – 94.4)

BMI: body mass index; BP: blood pressure; eGFR: estimated glomerular filtration rate; HDL: high-density lipoproteins; HOMA-IR: homeostasis model assessment of insulin resistance; HOMA-β: homeostasis model assessment of β-cell function; LDL: low-density lipoproteins; MDRD: modification of diet in renal disease study.

**Table 2:** **Metabolic characteristics of the cohort categorized according to the use of Diuretics**^**1**^

	**Use of Diuretics**		
	**No**	**Yes**	**P**^**2**^
	(n = 890)	(n = 71)	
Body weight (kg)	72.9 ± 15.5	82.8 ± 17.9	< 0.001
BMI (kg/m^2^)	27.5 ± 5.2	31.5 ± 6.2	< 0.001
Circumference:			
Waist (cm)	93.6 ± 13.8	102.4 ± 18.3	< 0.001
Hip (cm)	104.1 ± 11.7	110.0 ± 16.8	< 0.001
Systolic BP (mmHg)	129.1 ± 16.2	134.9 ± 17.7	0.005
Diastolic BP (mmHg)	78.6 ± 9.8	79.6 ± 9.6	0.22
Heart rate (beats/min)	74.3 ± 11.3	72.5 ± 11.6	0.18
Random capillary blood glucose (mg/dl)	87 ± 20	95 ± 23	0.001
Blood concentration of	n = 468	n = 39	
Glycated hemoglobin (%)	5.6 ± 0.43	5.8 ± 0.47	< 0.001
(mmol/mol)	37.2 ± 4.7	40.2 ± 5.2	
Glucose (mg/dL)	90 ± 11	99 ± 13	< 0.001
Total cholesterol (mg/dL)	213 ± 39	211 ± 43	0.69
HDL cholesterol (mg/dL)	60 ± 15	57 ± 12	0.52
Triglycerides (mg/dL)	100 ± 50	111 ± 35	0.008
LDL cholesterol (mg/dL)	134 ± 36	132 ± 38	0.71
Uric acid (mg/dL)	4.9 ± 1.4	5.8 ± 1.7	< 0.001
Insulin (μU/mL)	9.2 ± 5.5	13.2 ± 8.3	< 0.001
HOMA-I	2.10 ± 1.41	3.31 ± 2.43	< 0.001
Creatinine (mg/dL)	0.83 ± 0.21	0.87 ± 0.30	0.93
GFR - MDRD (mL/min/1.73 m^2^)	92.4 ± 19.1	90.8 ± 23.9	0.89

^1^ All data are reported as means ± SD.

^2^ Unpaired Student’s *t*-test.

BMI: body mass index; BP: blood pressure; GFR: glomerular filtration rate; HDL: high-density lipoproteins; HOMA-I: homeostasis model assessment of insulin resistance; LDL: low-density lipoproteins; MDRD: Modification of Diet in Renal Disease Study.

**Table 3:** Linear regression analysis of predictors of HOMA-IR

	**Outcome variable: HOMA-IR**		
**Parameter**	**Coefficient**	**Standard**	**P**
	**Estimate**	**Error**	**Chi-Squared**
Diet: healthy vs. non-healthy	−0.44	0.18	0.02
Diet: intermediate vs. non-healthy	−0.39	0.17	0.02
Gender: male vs. female	0.12	0.14	0.40
Use of diuretics: no vs. yes	−0.76	0.24	0.002
Use of beta-blockers: no vs. yes	0.13	0.22	0.56
Physical activity: none vs. moderate/heavy	0.47	0.19	0.02
Physical activity: light vs. moderate/heavy	0.23	0.19	0.23
Body mass index	0.07	0.02	<0.001
Waist circumference	0.02	0.006	<0.001
Age	−0.006	0.005	0.25

HOMA-IR: homeostasis model assessment of insulin resistance.

**Table 4:** Linear regression analysis of predictors of HOMA-β

	**Outcome variable: HOMA-β**		
**Variable**	**Coefficient**	**Standard**	**P**
	**Estimate**	**Error**	**Chi-Squared**
Diet: healthy vs. non-healthy	−17.36	13.35	0.19
Diet: intermediate vs. non-healthy	−20.31	12.30	0.098
Gender: male vs. female	−24.21	9.96	0.015
Use of diuretics: no vs. yes	8.34	17.76	0.64
Use of beta-blockers: no vs. yes	−21.33	16.24	0.19
Physical activity: none vs. moderate/heavy	19.70	14.08	0.16
Physical activity: light vs. moderate/heavy	25.53	14.08	0.070
Body mass index	3.99	1.21	<0.001
Waist circumference	0.03	0.05	0.46
Age	−1.72	0.04	<0.001

HOMA-β: homeostasis model assessment of β-cell function.

**Table 5:** Linear regression analysis of predictors of fasting plasma glucose concentrations

	**Outcome variable: Fasting plasma glucose**		
**Parameter**	**Coefficient**	**Standard**	**P**
	**Estimate**	**Error**	**Chi-Squared**
Diet: healthy vs. non-healthy	−0.17	14.41	0.91
Diet: intermediate vs. non-healthy	0.44	13.23	0.74
Gender: male vs. female	38.86	10.74	<0.001
Use of diuretics: no vs. yes	−62.59	19.19	0.001
Use of beta-blockers: no vs. yes	0.05	17.59	0.98
Physical activity: none vs. moderate/heavy	17.34	15.12	0.25
Physical activity: light vs. moderate/heavy	−0.56	15.17	0.71
Body mass index	0.15	0.13	0.26
Waist circumference	0.12	0.05	0.021
Age	0.14	0.04	<0.001

**Table 6:** Linear regression analysis of predictors of uric acid plasma concentrations

	**Outcome variable: Uric acid**		
**Parameter**	**Coefficient**	**Standard**	**P**
	**Estimate**	**Error**	**Chi-Squared**
Diet: healthy vs. non-healthy	0.01	0.16	0.93
Diet: intermediate vs. non-healthy	0.12	0.14	0.41
Sex: male vs. female	13.69	0.12	<0.001
Use of diuretics: no vs. yes	−0.53	0.21	0.01
Use of beta-blockers: no vs. yes	−0.27	0.19	0.15
Physical activity: none vs. moderate/heavy	−0.17	0.16	0.30
Physical activity: light vs. moderate/heavy	−0.10	0.16	0.55
Body mass index	0.023	0.014	0.11
Waist circumference	0.014	0.005	0.009
Age	0.01	0.04	0.03

**Table 7:** **Multivariate-adjusted odds ratios (OR) and 95**% **confidence intervals (CI) of pre-diabetes determined by diuretic use and other factors potentially associated with occurrence of Pre-diabetes**

**Effect**	**OR**^**1**^	**95% CI**
Age (y)	1.04	1.02 – 1.06
Gender (M vs. F)	0.68	0.44 – 1.05
Body Mass Index (kg/m^2^)	1.03	0.97 – 1.09
Waist Circumference (cm)	1.02	1.00 – 1.05
Dietary Pattern		
Intermediate vs. Mediterranean	1.17	0.74 – 1.85
Unhealthy vs. Mediterranean	1.32	0.73 – 2.37
Physical activity level:		
Light vs. none	0.87	0.56 – 1.36
Moderate/heavy vs. none	0.80	0.42 – 1.52
Use of Diuretics: yes vs. not	1.86	0.88 – 3.93
Use of Beta-blockers: yes vs. not	0.80	0.40 – 1.61

^1^The following baseline covariates were tested: age, gender (male vs. female), body mass index, waist circumference, dietary pattern (unhealthy, intermediate, Mediterranean), physical activity level (none, light and moderate/heavy), use of diuretics (yes vs. not), use of beta-blockers (yes vs. not).

## Discussion

This cross-sectional study suggests that the use of diuretics is independently associated with a diabetogenic metabolic pattern. First, insulin-resistance expressed as HOMA-IR was associated with the use of diuretics independent of other well-known influencing factors, such as diet, physical activity, BMI and waist circumference. Second, FPG, which is strongly influenced by neoglucogenesis, a biochemical pathway that is enhanced by insulin resistance, was independently associated with the use of diuretics. These results are of interest given that participants with known diabetes were excluded from the study. Abnormal glucose tolerance and hypertension are often associated in the context of the metabolic syndrome, and recognized as common features of insulin-resistance [1]. However, given the results of our multivariate analysis, we are inclined to exclude that the association of diuretic use with HOMA-IR and FPG is a consequence of the frequent association between diabetes and hypertension. Indeed, no other antihypertensive treatment, including beta-blockers, ACEI/ARBs and CAA, was associated with these variables. Also, we found that the frequency of previously unknown type 2 diabetes and pre-diabetes was significantly higher in those patients with hypertension who were on diuretic treatment than in those who were not, however, the use of diuretics was not independently associated with pre-diabetes. No association was found between diuretic use and HOMA-B, suggesting that the possible diabetogenic effect of these drugs does not involve the beta-cell function. Because the doses of diuretics habitually consumed by patients in our study were low, our results do not confirm those studies that found that low dosage diuretics have no significant effect on glucose homeostasis [10,28]. Among the mechanisms by which diuretics can induce insulin resistance, hypokalemia consequent to diuretics use has been considered principally responsible for impaired insulin sensitivity [7,28] despite the fact that the unfavorable effect on glucose homeostasis persists, even if mitigated, when oral potassium is supplemented [29]. Unfortunately, serum potassium concentrations were not measured in this study.

Our study also confirms that the use of diuretics is independently associated with uric acid levels, a well-known effect in connection with insulin resistance. Elevated serum uric acid levels are commonly seen in association with glucose intolerance, hypertension and dyslipidemia, a cluster of metabolic and hemodynamic disorders that characterizes the metabolic syndrome [30-34]. Hyperinsulinemic, insulin-resistant people have a decreased clearance of uric acid in the renal proximal tubule that is not insulin resistant [35]. However, other mechanisms may explain the hyperuricemic effect of diuretics. Volume depletion consequent to diuretic treatment reduces renal blood flow, with consequent urate underexcretion. Indeed, diuretics influencing the ion exchange proteins at the proximal tubule lumen membrane in the kidney increase both sodium and urate reabsorption [36]. Despite the fact that the role of uric acid as an independent contributor to cardiovascular risk remains uncertain [13,37], different mechanisms induced by uric acid have been proposed as agents that may be responsible for unfavorable cardiovascular effects, including enhanced platelet aggregation [38], inflammation and endothelial dysfunction [39]. The association between use of diuretics and serum uric acid concentrations is not of secondary importance. Treatment of hypertension with diuretics raises uric acid concentrations, though there is evidence that increased uric acid concentrations may themselves contribute to inducing hypertension [37]. Therefore, the relationship between diuretic use, hypertension, insulin-resistance and abnormal glucose tolerance may be more complex, and it is possible that diuretic treatment may partially offset the benefits of reduction of blood pressure. Kivity et al. [40] recently reported that after a follow-up of 4.8 years, serum uric acid was independently associated with cardiovascular disease in healthy people, especially in women. In the WORKSITE study, an increase in serum uric acid was independently associated with cardiovascular events [41]. Moreover, in the SHEP trial, the reduction in coronary events through treatment with a diuretic was not be observed when serum uric acid increased more than 60 μmol/L during treatment [42]. These studies suggest that hyperuricemia consequent to diuretic use may, in part, offset the benefits of diuretics in preventing cardiovascular complications of hypertension.

Despite these findings, our study should be considered with caution given that diuretics have proven extremely useful in the prevention of stroke and cardiovascular events in both diabetics and non-diabetics [42,43]. Also, diuretics did not increase the risk of diabetes in a longitudinal observation that included 458 patients [44]. This study has several limitations. First, a larger cohort may have allowed for more robust conclusions. The sample size was relatively small, and only about 53% of the cohort had complete laboratory measurements, this may have blunted the statistical power of the observed associations. Given the cross-sectional design of the study, we cannot exclude the possibility of residual confounding. We did not perform an oral glucose tolerance test, which is the gold standard for classifying individual glucose tolerance levels [24], thus possibly underestimating the frequency of pre-diabetes and diabetes in our cohort. Nonetheless, the combined use of FPG and HbA_1_c (we also included random glycemia) is acknowledged as a sensitive and specific screening tool for identifying individuals with diabetes and impaired glucose tolerance [45,46]. We did not enroll a representative cohort of the Palermo population and some bias might be associated with the sampling technique. However, the composition of the cohort we recruited was similar to that reported for the shopping mall customers. In addition, having also offered the possibility of a thyroid echography check likely prompted younger people without known cardiovascular, metabolic or nutritional clinical problems to take part in the study. More women than men participated, though this is a common problem in all trials, screening and epidemiological studies. Our study does, however, have some merits. This is a single center study, and the modality of participant recruitment likely allowed for the selection of a cohort that was representative of the population. Due to the small number of participants, we were not able to distinguish the effects of each single category of diuretics, and thus have to propose a unique class effect. Indeed, for the same reasons, we could not distinguish differences among different doses of diuretics. However, our study also has the merit of having considered both the effects on uric acid concentrations and insulin resistance. It is worth noting that we measured all variables potentially influencing the outcomes of the study, including anthropometric measurements, dietary factors and habitual physical activity. In addition, the enrollment of participants occurred within a short time, in the same season, and a small, select group of dieticians administered questionnaires face-to-face, all of which may have contributed to increasing the quality of the data.

## Conclusions

We can confirm that use of diuretics, even at low dosages, is associated with insulin resistance and increased serum uric acid levels in adults without known diabetes and/or cardiovascular diseases. We also found evidence that abnormal glucose tolerance is associated with use of diuretics. However, only longitudinal interventional studies can elucidate the effective influence of diuretics on global cardiovascular risk.

## Abbreviations

ACEI: Angiotensin converting enzyme inhibitors; ARBs: Angiotensin receptor blockers; BMI: Body mass index; BP: Blood pressure; CCA: Calcium channel antagonists; FFQ: Food frequency questionnaire; FPG: Fasting plasma glucose; HbA1c: Glycated hemoglobin; HDL: High density lipoproteins; HOMA-β: Homeostasis model assessment of β-cell function; HOMA-IR: Homeostasis model assessment of insulin resistance; LDL: Low density lipoproteins.

## Competing interests

The authors declare that they have no competing interests.

## Authors’ contributions

The authors’ responsibilities were as follows: SB conceived of the study, participated in its design and coordination, analyzed and interpreted data, and drafted the manuscript. AN analyzed data and interpreted and contributed to preparation of the manuscript. FMM performed the laboratory tests and revised the manuscript. DS collected data and helped draft the manuscript. GL analyzed data, and revised the manuscript. FG interpreted data and revised the manuscript. EA analyzed data and revised the manuscript. GG interpreted data, and revised the manuscript. AB recruited volunteers, managed the clinical study, carried out anthropometric measurements, collected data, and drafted the manuscript. GBR was responsible for the study, and revised the manuscript critically for important intellectual content. All authors read and approved the final manuscript.

## References

[B1] National Institute of Health National Institute of Diabetes and Digestive and Kidney Diseases: Diabetes in America 1995 2 Bethesda, US: National Institute of Health

[B2] Epstein M Sowers JR Diabetes mellitus and hypertension Hypertension 1992 5 403 418 1568757 10.1161/01.hyp.19.5.403

[B3] Aromaa A Reunanen A Pyorala K Hypertension and mortality in diabetic and non-dibetic Finnish men J Hypertens 1984 5 205 207 6599669

[B4] Eriksson JW Jansson PA Carlberg B Hagg A Kurland L Svensson MK Ahlstrom H Strom C Lonn L Ojbrandt K Johansson L Lind L Hydrochlorothiazide, but not candesartan, aggravates insulin resistance and causes visceral and hepatic fat accumulation Hypertension 2008 5 1030 1037 18981327 10.1161/HYPERTENSIONAHA.108.119404

[B5] Stears AJ Woods SH Watts MM Burton TJ Graggaber J Mir FA Brown MJ A double-blind, placebo controlled, crossover trial comparing the effects of Amiloride and hydrochlorothiazide on glucose tolerance in patients with essential hypertension Hypertension 2012 5 934 942 22493073 10.1161/HYPERTENSIONAHA.111.189381

[B6] Elliott WJ Meyer PM Incident diabetes in clinical trials of antihypertensive drugs: a network meta-analysis Lancet 2007 5 201 207 17240286 10.1016/S0140-6736(07)60108-1

[B7] Meisinger C Stockl D Ruckert IM Doring A Thorand B Heier M Huth C Belcredi P Kowall B Rathmann W Serum potassium is associated with prediabetes and newly diagnosed diabetes in hypertensive adults from the general population: the KORA F4-study Diabetologia 2013 5 484 491 23183943 10.1007/s00125-012-2786-8

[B8] Lundgren H Bjorkman L Keiding P Lundmark S Bengtsson C Diabetes in patients with hypertension receiving pharmacological treatment BMJ 1988 5 1512 2906258 10.1136/bmj.297.6662.1512 PMC1835198

[B9] Bengtsson C Blohmé C Lapidus L Lundgren H Diabetes in hypertensive women: an effect of antihypertensive drugs or the hypertensive state per se ? Diabet Med 1988 5 261 264 2967148 10.1111/j.1464-5491.1988.tb00981.x

[B10] Savage PJ Pressel SL Curb JD Schron EB Applegate WB Black HR Cohen J Davis BR Frost P Smith W Gonzalez N Guthrie GP Oberman A Rutan G Probstfield JL Stamler J Influence of long-term, low dose, diuretic based, antihypertensive therapy on glucose, lipid, uric acid, and potassium levels in older men and women with isolated systolic hypertension Arch Intern Med 1998 5 741 751 9554680 10.1001/archinte.158.7.741

[B11] ALLHAT Officers and Coordinators for the ALLHAT Collaborative Research Group The Antihypertensive and Lipid-Lowering Treatment to Prevent Heart Attack Trial: Major outcomes in high-risk hypertensive patients randomized to angiotensin-converting enzyme inhibitor or calcium channel blocker vs diuretic: The Antihypertensive and Lipid-Lowering Treatment to Prevent Heart Attack Trial (ALLHAT) JAMA 2002 5 2981 2997 10.1001/jama.288.23.2981 12479763

[B12] McAdams DeMarco MA Maynard JW Baer AN Gelber AC Young JH Alonso A Coresh J Diuretic use, increased serum urate levels, and risk of incident gout in a population-based study of adults with hypertension Arthritis Rheum 2012 5 121 129 22031222 10.1002/art.33315 PMC3253199

[B13] Franse LV Pahorb M Di Baric M Shorrd RI Wand JY Somesd GW Applegatee WB Serum uric acid, diuretic treatment and risk of cardiovascular events in the Systolic Hypertension in the Elderly Program (SHEP) J Hypertens 2000 5 1149 1154 10954008 10.1097/00004872-200018080-00021

[B14] Vijan S Hayward RA Treatment of hypertension in type 2 diabetes mellitus: blood pressure goals, choice of agents, and setting priorities in diabetes care Ann Intern Med 2003 5 593 602 12667032 10.7326/0003-4819-138-7-200304010-00018

[B15] Gu Q Burt VL Dillon CF Yoon S Trends in antihypertensive medication use and blood pressure control among United States adults with hypertension: the National Health and Nutrition Examination Survey, 2001 to 2010 Circulation 2012 5 2105 2114 23091084 10.1161/CIRCULATIONAHA.112.096156

[B16] Rosendorff C Black HR Cannon CP Gersh BJ Gore J Izzo JL Kaplan NM O’Connor CM O’Gara PT Oparil S Treatment of hypertension in the prevention and management of ischemic heart disease - a scientific statement from the American Heart Association Council for High Blood Pressure Research and the Councils on Clinical Cardiology and Epidemiology and Prevention Circulation 2007 5 2761 2788 17502569 10.1161/CIRCULATIONAHA.107.183885

[B17] Mancia G De Backer G Dominiczak A Cifkova R Fagard R Germano G Grassi G Heagerty AM Kjeldsen SE Laurent S Guidelines for the management of arterial hypertension: The Task Force for the Management of Arterial Hypertension of the European Society of Hypertension (ESH) and of the European Society of Cardiology (ESC) Eur Heart J 2007 5 1462 1536 17562668 10.1093/eurheartj/ehm236

[B18] Cooper-DeHoff RM Pacanowski MA Pepine CJ Cardiovascular therapies and associated glucose homeostasis: implications across the dysglycemia continuum J Am Coll Cardiol 2009 5 28 34 10.1016/j.jacc.2008.10.037 PMC2655143 19179214

[B19] Fujiwara W Izawa H Ukai G Yokoi H Mukaide D Kinoshita K Morimoto SI Ishii J Ozaki Y Nomura M Low dose hydrochlorothiazide, in combination with angiotensin receptor blocker, reduces blood pressure effectively without adverse effect on glucose and lipid profiles Heart Vessels 2012 5 22447467 10.1007/s00380-012-0246-5 22447467

[B20] Bakris G Molitch M Hewkin A Kipnes M Sarafidis P Fakouhi K Bacher P Sowers J Differences in glucose tolerance between fixed-dose antihypertensive drug combinations in people with metabolic syndrome Diabetes Care 2006 5 2592 2597 17130190 10.2337/dc06-1373

[B21] Sowers JR Raij L Jialal I Egan BM Ofili EO Samuel R Zappe DH Purkayastha D Deedwania PC Angiotensin receptor blocker/diurectic combination preserves insulin responses in obese hypertensives J Hypertens 2010 5 176 179 10.1097/HJH.0b013e32833af380 PMC2908201 20498618

[B22] Willett W Nutrition Epidemiology 1988 2 New York: Oxford University Press

[B23] Buscemi S Nicolucci A Mattina A Rosafio G Massenti FM Lucisano G Galvano F Amodio E Pellegrini E Barile AM Maniaci V Grosso G Verga S Sprini D Rini GB Association of dietary patterns with insulin resistance and clinically silent carotid atherosclerosis in apparently healthy people Eur J Clin Nutr 2013 5 1284 1290 24045794 10.1038/ejcn.2013.172

[B24] American Diabetes Association Diagnosis and classification of diabetes mellitus Diabetes Care 2013 5 67 74

[B25] Friedewald WT Estimation of the concentration of low density lipoprotein cholesterol in plasma, without use of the preparative ultracentrifuge Clin Chem 1972 5 499 502 4337382

[B26] Rule AD Larson TS Bergstralh EJ Slezak JM Jacobsen SJ Cosio FG Using serum creatinine to estimate glomerular filtration rate: accuracy in good health and in chronic kidney disease Ann Intern Med 2004 5 929 937 15611490 10.7326/0003-4819-141-12-200412210-00009

[B27] Matthews DR Hosker JP Rudenski AS Naylor BA Treacher DF Turner RC Homeostasis model assessment: insulin resistance and b-cell function from fasting plasma glucose and insulin concentrations in man Diabetologia 1985 5 412 419 3899825 10.1007/BF00280883

[B28] Salvetti A Ghiadoni L Thiazide diuretics in the treatment of hypertension: an update J Am Soc Nephrol 2006 5 25 29 10.1681/ASN.2005121329 16565243

[B29] Zillich AJ Garg J Basu S Bakris GL Carter BL Thiazide diuretics, potassium, and the development of diabetes Hypertension 2006 5 219 224 16801488 10.1161/01.HYP.0000231552.10054.aa

[B30] Bonora E Targher G Zenere MB Saggiani F Cacciatori V Tosi F Travia D Zenti MG Branzi P Santi L Muggeo M Relationship of uric acid concentration to cardiovascular risk factors in young men. The role of obesity and central fat distribution. The Verona young Men atherosclerosis risk factors study Int J Obes Relat Metab Disord 1996 5 975 980 8923153

[B31] Zavaroni I Mazza S Fantuzzi M Dall’Aglio E Bonora E Delsignore R Passeri M Reaven GM Changes in insulin and lipid metabolism in males with asymptomatic hyperuricemia J Intern Med 1993 5 25 30 8326285 10.1111/j.1365-2796.1993.tb00700.x

[B32] Vuorinen-Markkola H Yki-Järvinen H Hyperuricemia and insulin resistance J Clin Endocrinol Metab 1994 5 25 29 8288709 10.1210/jcem.78.1.8288709

[B33] Facchini F Ida Chen Y-D Hollenbeck CB Reaven GM Relationship between resistance to insulin-mediated glucose uptake, urinary uric acid clearance and plasma uric acid concentration JAMA 1991 5 3008 3011 1820474

[B34] Reaven GM The kidney: an unwilling accomplice in syndrome X Am J Kidney Dis 1997 5 928 931 9398143 10.1016/s0272-6386(97)90106-2

[B35] Rathmann W Funkhouser E Dyer AR Roseman JM Relations of hyperuricemia with the various components of the insulin resistance syndrome in young black and white adults: the CARDIA study. Coronary artery risk development in young adults Ann Epidemiol 1998 5 250 261 9590604 10.1016/s1047-2797(97)00204-4

[B36] Johnson RJ Kang DH Feig D Kivlighn S Kanellis J Watanabe S Tuttle KR Rodriguez-Iturbe B Herrera-Acosta J Mazzali M Is there a pathogenetic role for uric acid in hypertension and cardiovascular and renal disease? Hypertension 2003 5 1183 1190 12707287 10.1161/01.HYP.0000069700.62727.C5

[B37] Feig DI Kang DH Johnson RJ Uric acid and cardiovascular risk N Engl J Med 2008 5 1811 1821 18946066 10.1056/NEJMra0800885 PMC2684330

[B38] Jaques BC Ginsberg MH The role of cell surface proteins in platelet stimulation by monosodium urate crystals Arthritis Rheum 1982 5 508 521 7082398 10.1002/art.1780250504

[B39] Chapman PT Yarwood H Harrison AA Stocker CJ Jamar F Gundel RH Peters AM Haskard DO Endothelial activation in monosodium urate monohydrate crystal-induced inflammation: in vitro and in vivo studies on the roles of tumor necrosis factor alpha and interleukin-1 Arthritis Rheum 1997 5 955 965 9153559 10.1002/art.1780400525

[B40] Kivity S Kopel E Maor E Abu-BAchar F Segev S Sidi Y Olchovsky D Association of serum uric acid and cardiovascular disease in healthy adults Am J Cardiol 2013 5 1146 1151 23352265 10.1016/j.amjcard.2012.12.034

[B41] Alderman MH Cohen H Madhavan S Kivlighn S Serum uric acid and cardiovascular events in successfully treated hypertensive patients Hypertension 1999 5 144 150 10406838 10.1161/01.hyp.34.1.144

[B42] Chobanian AV Bakris GL Black HR Cushman WC Green LA Izzo JL Jr Jones DW Materson BJ Oparil S Wright JT Jr Roccella EJ for the National Heart, Lung, and Blood Institute Joint National Committee on Prevention, Detection, Evaluation, and Treatment of High Blood Pressure; National High Blood Pressure Education Program Coordinating Committee The seventh report of the Joint National Committee on Prevention, Detection, Evaluation, and Treatment of High Blood Pressure: the JNC 7 report JAMA 2003 5 2560 257212748199

[B43] Whelton PK Barzilay J Cushman WC Davis BR Iiamathi E Kostis JB Leenen FH Louis GT Margolis KL Mathis DE Moloo J Nwachuku C Panebianco D Parish DC Pressel S Simmons DL Thadani U Clinical outcomes in antihypertensive treatment of type 2 diabetes, impaired fasting glucose concentration, and normoglycemia: Antihypertensive and Lipid-Lowering Treatment to Prevent Heart Attack Trial (ALLHAT) Arch Intern Med 2005 5 1401 1409 15983290 10.1001/archinte.165.12.1401

[B44] Gress TW Nieto J Shahar E Wofford MR Brancati FL Hypertension and antihypertensive therapy as risk factors for type 2 diabetes mellitus N Engl J Med 2000 5 905 912 10738048 10.1056/NEJM200003303421301

[B45] Kim KS Kim SK Lee YK Park SW Cho YW Diagnostic value of glycated haemoglobin HbA (1c) for the early detection of diabetes in high-risk subjects Diabet Med 2008 5 997 1000 18959616 10.1111/j.1464-5491.2008.02489.x

[B46] Hu Y Liu W Chen Y Zhang M Wang L Zhou H Wu P Teng X Dong Y Zhou J Xu H Zheng J Li S Tao T Hu Y Jia Y Combined use of fasting plasma glucose and glycated hemoglobin A1c in the screening of diabetes and impaired glucose tolerance Acta Diabetol 2010 5 231 236 19760291 10.1007/s00592-009-0143-2

